# ﻿Taxonomic notes on the genus *Spinosodus* Breuning & de Jong, 1941 (Coleoptera, Cerambycidae) with a generic and specific synonym

**DOI:** 10.3897/zookeys.1223.137172

**Published:** 2025-01-17

**Authors:** Shuai Zhao, Ting Qin, Guanglin Xie, Wenkai Wang

**Affiliations:** 1 Institute of Entomology, College of Agriculture, Yangtze University, Jingzhou, Hubei, 434025, China; 2 Guangxi Forestry Inventory and Planning Institute, Nanning, Guangxi, 530011, China; 3 Hubei Engineering Research Center for Pest Forewarning and Management, Yangtze University, Jingzhou, Hubei, 434025, China

**Keywords:** *Bulbolmotega*, Lamiinae, new record, new synonym

## Abstract

Taxonomic notes on the genus *Spinosodus* Breuning & de Jong, 1941 are presented. The genus *Bulbolmotega* Breuning, 1966 is synonymized with *Spinosodus*, and *Bulbolmotegasumatrensis* Breuning, 1966 is recognized as a junior synonym of *Spinosodusspinicollis* Breuning & de Jong, 1941. Additionally, *Spinosodus* is redescribed, and *S.rufomaculatus* Breuning, 1973 is formally reported from China, Vietnam, Thailand, and India for the first time.

## ﻿Introduction

The genus *Spinosodus* Breuning & de Jong, 1941 (Coleoptera, Cerambycidae) was established for *Spinosodusspinicollis* Breuning & de Jong, 1941 from Java, Indonesia. Subsequently, both the genus and the species were redescribed by Breuning (1963) in a revision of the Asian Pteropliini. The second species of this genus, *Spinosodusrufomaculatus* Breuning, 1973 was described based on a single specimen from Pakson, Laos. Currently, the genus is recognized as comprising these two species: the insular *S.spinicollis* from Indonesia and the continental *S.rufomaculatus* from Laos (Tavakilian and Chevillotte 2024). The monotypic genus *Bulbolmotega* Breuning, 1966 was described for *Bulbolmotegasumatrensis* Breuning, 1966 from Sumatra (Indonesia), and no additional species are known to date. Recently, two intriguing individuals of these genera were collected in Guangxi Zhuang Autonomous Region, China. Initial identification suggested that these specimens belong to *Spinosodus*. However, further comparison revealed striking similarities to *Bulbolmotega*. This observation triggered us to discuss the relationship between these two genera. Therefore, we aim to verify whether the genus *Bulbolmotega* should be synonymized with *Spinosodus*, based on a comparison of the type material.

## ﻿Materials and methods

Specimens from the following collections were examined and/or photographed in this study. The place where the specimens were deposited is indicated in the text.

**MNHN**Muséum National d’Histoire Naturelle, Paris, France;

**RMNH**Rijksmuseum van Natuurlijke Historie, Leiden, Holland;

**SNSD** Senckenberg Naturhistorische Sammlungen Dresden, Dresden, Germany;

**YZU** Yangtze University, Jingzhou, China;

**CXG** Collection of Xavier Gouverneur, Rennes, France.

The photographs of the specimens from Guangxi were taken using a Canon 7D Mark II digital camera equipped with a Canon EF 100 mm f/2.8L IS USM. The photographs of the holotype of *S.spinicollis* were photographed by Oscar Vorst (RMNH). The photographs of the holotype of *S.rufomaculatus* were photographed by Antoine Mantilleri and Christophe Rivier (MNHN). The photographs of the holotype of *B.sumatrensis* were photographed by Olaf Jäger (SNSD). All photographs were edited using Adobe Photoshop 2020.

## ﻿Results

After examining photographs of the type specimens of both genera, it is clear that the genus *Bulbolmotega* should be considered a junior synonym of *Spinosodus*. Consequently, *B.sumatrensis* is recognized as a junior synonym of *S.spinicollis*. Additionally, *S.rufomaculatus* is formally reported here for the first time from China, Vietnam, Thailand, and India based on own observations and the data presented on the Cerambycoidea Forum (Vitali 2024), as well as the materials provided by Xavier Gouverneur.

### ﻿Genus Spinosodus

Taxon classificationAnimaliaColeopteraCerambycidae

Breuning & de Jong, 1941

3F9DD8B1-48DA-59A1-BE19-5436FFE5E714

Spinosodus Breuning & de Jong, 1941: 96—Breuning 1961: 283; Breuning 1963: 509. Type species: S.spinicollis Breuning & de Jong, 1941.
Bulbolmotega Breuning, 1966: 124. Type species: B.sumatrensis Breuning, 1966. Syn. nov.

#### Redescription.

Body relatively broad. Head retracted backwards, frons wider than long. Antennae shorter than body, with short setae beneath; antennal insertions flat, not obviously protruding upwards, widely separated from each other; scape short and stout, pedicel relatively long, antennomere 3 slightly longer than antennomere 4 or scape. Eyes slightly coarsely faceted, inner side deeply emarginate, lower lobe longer than broad, remarkably longer than gena. Pronotum transverse, with two transverse grooves on anterior and posterior margins, respectively, the second one on anterior margin strongly curved backwards at middle; each side provided a small but acute spine behind the middle, slightly directed backwards; disc uneven, with a large and blunt hump on each side. Elytra wider than pronotum, rounded apically; each elytron with a longitudinal blunt median ridge at base. Prosternal process narrow, lower than procoxae; mesosternal process shortly vertical anteriorly; metasternum normal. Legs moderately long, femur slightly clavate.

#### Comments.

Based on the comparison of the type species of *Spinosodus* and *Bulbolmotega* (images and original descriptions), we found no significant morphological differences between these genera. As a result, *Bulbolmotega* is considered a junior synonym of *Spinosodus*.

### ﻿ Spinosodus spinicollis

Taxon classificationAnimaliaColeopteraCerambycidae

Breuning & de Jong, 1941

31B8D61A-B619-5876-B21E-92E351577F61

**Figure 1.**
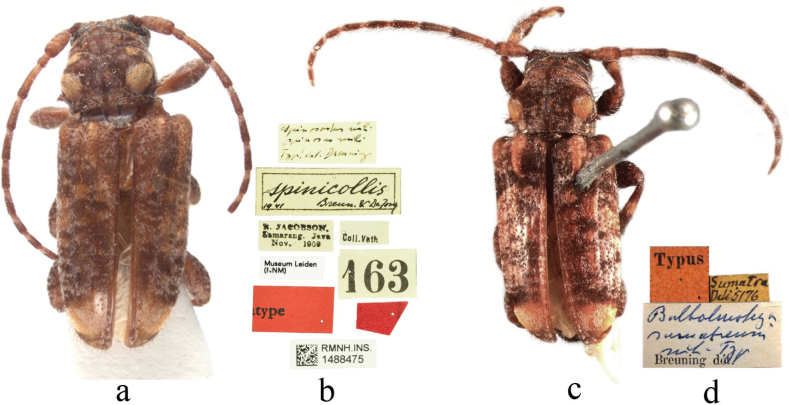
Habitus of *Spinosodusspinicollis* Breuning & de Jong, 1941 **a, b** holotype of *S.spinicollis***c, d** holotype of *Bulbolmotegasumatrensis* Breuning, 1966.

Figs 1 , 3a–d , 4a, b, e, f

Spinosodus spinicollis Breuning & de Jong, 1941: 96—Breuning 1961: 283; Breuning 1963: 509. Type locality: Java, Indonesia.
Bulbolmotega sumatrensis Breuning, 1966: 124. Type locality: Sumatra, Indonesia. Syn. nov.

#### Type material examined.

***Holotype*** of *S.spinicollis* (RNMH, INS. 1488475), label details are shown in Fig. 1b. ***Holotype*** of *B.sumatrensis* (SNSD), label details are shown in Fig. 1d.

#### Distribution.

Indonesia.

#### Comments.

Based on the comparison of *S.spinicollis* and *B.sumatrensis*, we found that there are no significant differences between the two species. For example, in both species, the body colour is predominantly pale reddish brown, the pronotum has a small, rounded, ochraceous pubescent patch on each side of the anterior margin, which is distinctly separated from the outer pubescent patch and the posterior large, rounded pubescent patch, and the elytra that are unevenly scattered with the yellowish brown pubescence. Therefore, we propose that *B.sumatrensis* Breuning, 1966 is a junior synonym of *S.spinicollis* Breuning & de Jong, 1941.

As *B.sumatrensis* is the type species of *Bulbolmotega*, this genus becomes a junior synonym of the genus *Spinosodus*.

### ﻿ Spinosodus rufomaculatus

Taxon classificationAnimaliaColeopteraCerambycidae

Breuning, 1973

DC722425-53EB-562B-A0DB-87BCFFBB291B

**Figure 2.**
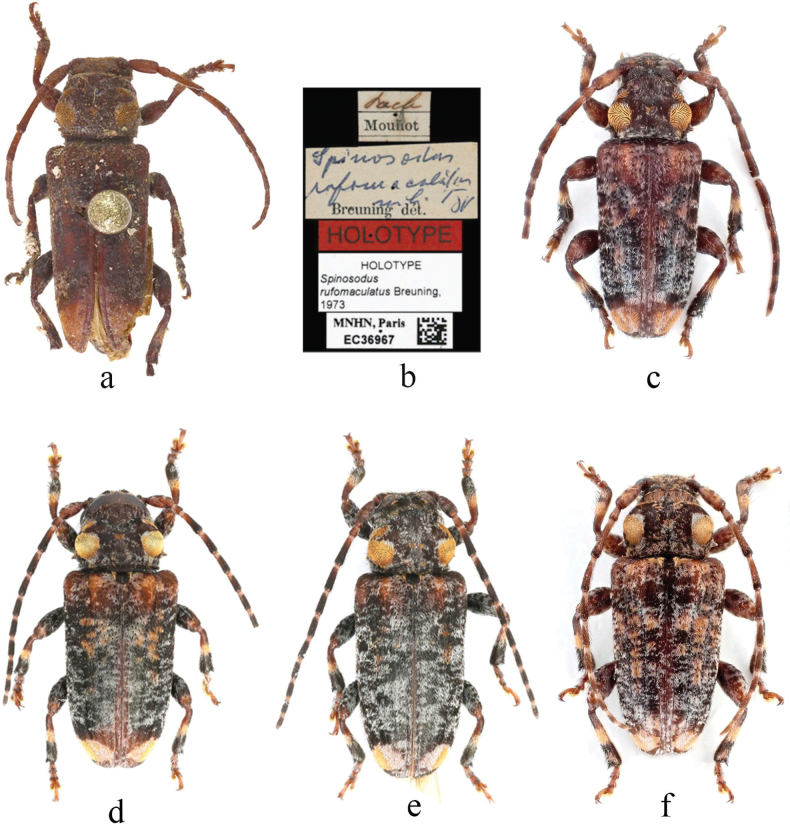
Habitus of *Spinosodusrufomaculatus* Breuning, 1973 **a, b** holotype **c** the individual from Oudomxay, Laos **d, e** individuals from Guangxi, China **f** individual from Phrae, Thailand.

**Figure 3.**
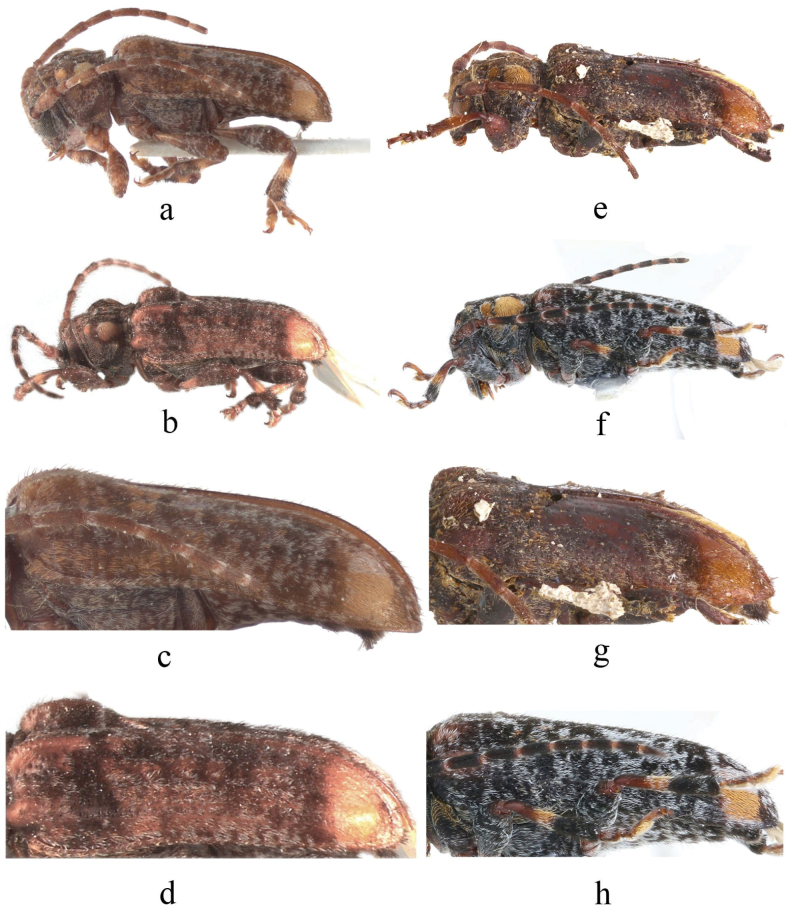
Habitus of *Spinosodus* spp. **a, c** holotype of *S.spinicollis* Breuning & de Jong, 1941 **b, d** holotype of *Bulbolmotegasumatrensis* Breuning, 1966 **e–h***Spinosodusrufomaculatus* Breuning, 1973 **e, g** holotype **f, h** individual from Guangxi, China.

**Figure 4.**
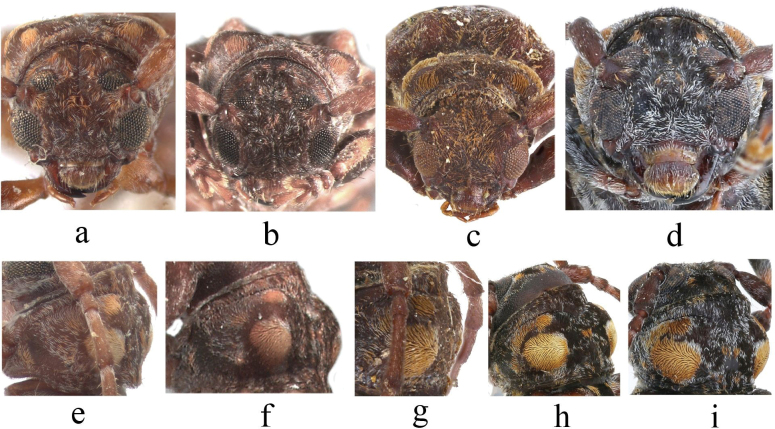
Habitus of *Spinosodus* spp. **a, e** holotype of *Spinosodusspinicollis* Breuning & de Jong, 1941 **b, f** holotype of *Bulbolmotegasumatrensis* Breuning, 1966 **c, d, g–i***Spinosodusrufomaculatus* Breuning, 1973 **c, g** holotype **d, h, i** individuals from Guangxi, China.

Figs 2 , 3e–h , 4c, d, g–i

Spinosodus rufomaculatus Breuning, 1973: 660.

#### Type material examined.

***Holotype*** (MNHN, EC36967), label details are shown in Fig. 2b.

#### Non-type material examined.

**China** • 2 females (YZU); Guangxi, Fengshan county, Fengcheng town; 24°23'56.78"N, 107°1'30.46"E; alt. 479 m; 24 Apr. 2024; Yitong Fu leg.; captured by light trap • **Laos**: 1 male (CXG); Oudomxay province, Nam Kat; alt. 750 m; May 2024; Steeve Collard leg.; captured by UV light trap • **Thailand**: 1 female (CXG); Phrae province, Wangchin, Punjen; alt. 436 m; 16 Apr. 2017; Xavier Gouverneur leg.; captured by UV light trap.

#### Distribution.

Laos, China (new country record), Vietnam (new country record), Thailand (new country record), and India (new country record).

#### Comments.

This species is very similar to the type species, *S.spinicollis* from Indonesia, with the main differences being in body coloration and the shape of pubescent patches on the pronotum. In *S.rufomaculatus*, the body color is darker, and the premedian pubescent patches on both sides of the pronotum are narrow, transverse, and uniformly ochre-colored, while in the type species, the body color is lighter, and the premedian pubescent patches on the pronotum are nonuniform in color, featuring a distinct, circular ochre spot on the inner side with noticeably lighter pubescence on the outer side.

Previously, *S.rufomaculatus* was known only from Laos. However, based on our own records presented in this study (Fig. 2d, e), data and images provided by Xavier Gouverneur (Fig. 2c, f), and the Cerambycoidea Forum (Vitali 2024), the range of this species has been extended to Guangxi and Yunnan in China, Cao Bang in Vietnam, Phrae in Thailand, and Kerala in India.

## ﻿Discussion

Breuning and de Jong (1941) described *Spinosodusspinicollis* based on the specimen from Java, Indonesia. Subsequently, the same author (Breuning 1966) described *Bulbolmotegasumatrensis* from Sumatra, Indonesia. Although Breuning stated in the original description of *B.sumatrensis* that the lower eye lobe is about five times as long as the gena, compared to four times in *S.spinicollis*, we found that the difference most likely due to a measurement error. In fact, there are no taxonomically significant differences between the two species.

*Spinosodusspinicollis* is highly similar to *S.rufomaculatus* in external habitus, differing primarily in body color and the shape and color of the premedian pubescent patches on the pronotum. However, the available material indicates that *S.rufomaculatus* shows some intraspecific variability in body color and pubescence distribution, which suggests that the coloration and pubescent patterns are not reliable characters for differentiation of these two taxa. Nevertheless, we treat them here as separate species due to their clearly different geographic ranges. The type species is insular, found in Java, Sumatra, and Borneo in Indonesia, while *S.rufomaculatus* is a typical continental species, recorded from Laos, China, Vietnam, Thailand, and India.

The genus *Spinosodus* was originally placed in tribe Pteropliini Thomson, 1860, while the genus *Bulbolmotega* was classified in the tribe Acanthocinini Blanchard, 1845. However, we show that the mesotibia lacks an external oblique groove near the apex and the mesocoxal cavity opens laterally, which suggests that *Spinosodus* belongs to Pteropliini rather than Acanthocinini. Breuning and de Jong (1941) indicated that *Spinosodus* is closely related to *Sodus* Pascoe, 1865 (= *Similosodus* McKeown, 1945), but it differs from the latter in the antennae, which are distinctly shorter than the body, and in the pronotum, which is equipped with a short, small lateral spine located posterior to the middle of each side.

## Supplementary Material

XML Treatment for Spinosodus

XML Treatment for Spinosodus spinicollis

XML Treatment for Spinosodus rufomaculatus

## ﻿Additional information

### Author ORCIDs

Shuai Zhao https://orcid.org/0000-0002-1021-9060

Guanglin Xie https://orcid.org/0000-0003-3273-8985

### Data availability

All of the data that support the findings of this study are available in the main text.
